# Spatiotemporal analysis of cutaneous leishmaniasis in Palestine and foresight study by projections modelling until 2060 based on climate change prediction

**DOI:** 10.1371/journal.pone.0268264

**Published:** 2022-06-09

**Authors:** Ahmad Amro, Olga Moskalenko, Omar Hamarsheh, Marcus Frohme

**Affiliations:** 1 Faculty of Pharmacy, Al-Quds University, Abu-Dies, Jerusalem, Palestine; 2 Molecular Biotechnology and Functional Genomics, Technical University of Applied Sciences Wildau, Wildau, Germany; 3 BIOMES NGS GmbH, Wildau, Germany; 4 Department of Biological Sciences, Faculty of Science and Technology, Al-Quds University, Abu-Dies, Jerusalem, Palestine; Iran University of Medical Sciences, ISLAMIC REPUBLIC OF IRAN

## Abstract

**Background:**

Cutaneous leishmaniasis (CL) is a vector-borne parasitic diseases of public health importance that is prevalent in the West Bank but not in the Gaza Strip. The disease caused by parasitic protozoans from the genus *Leishmania* and it is transmitted by infected phlebotomine sand flies. The aim of our study is to investigate the eco-epidemiological parameters and spatiotemporal projections of CL in Palestine over a 30-years period from 1990 through 2020 and to explore future projections until 2060.

**Methodology/Principal findings:**

This long-term descriptive epidemiological study includes investigation of demographic characteristics of reported patients by the Palestinian Ministry of Health (PMoH). Moreover, we explored spatiotemporal distribution of CL including future projection based on climate change scenarios. The number of CL patients reported during this period was 5855 cases, and the average annual incidence rate (AAIR) was 18.5 cases/10^5^ population. The male to female ratio was 1.25:1. Patients-age ranged from 2 months to 89 years (mean = 22.5, std 18.67, and the median was 18 years). More than 65% of the cases came from three governates in the West Bank; Jenin 29% (1617 cases), Jericho 25% (1403), and Tubas 12% (658) with no cases reported in the Gaza Strip. Seasonal occurrence of CL starts to increase in December and peaked during March and April of the following year. Current distribution of CL indicate that Jericho, Tubas, Jenin and Nablus have the most suitable climatic settings for the sandfly vectors. Future projections until 2060 suggest an increasing incidence from northwest of Jenin down to the southwest of Ramallah, disappearance of the foci in Jericho and Tubas throughout the Jordan Vally, and possible emergence of new foci in Gaza Strip.

**Conclusions/Significance:**

The future projection of CL in Palestine until 2060 show a tendency of increasing incidence in the north western parts of the West Bank, disappearance from Jericho and Tubas throughout the Jordan Vally, and emergence of new CL endemic foci in the Gaza Strip. These results should be considered to implement effective control and surveillance systems to counteract spatial expansion of CL vectors.

## Introduction

Leishmaniasis is a neglected tropical disease caused by an obligatory intracellular protozoan parasite belonging to the genus *Leishmania* and transmitted to humans by the bite of bloodsucking sand-flies of the genus *Phlebotomus* in the Old World and *Lutzomyia* in the New World. The three main clinical pictures of leishmaniasis are cutaneous leishmaniasis (CL), which is the most common, mutilating mucocutaneous, and visceral leishmaniasis, which can be lethal if left untreated. The disease has a wide distribution and is endemic in circumscribed areas in Asia, Africa, Europe, Middle East, and Central and South America [1, 2].

Cutaneous Leishmaniases continues to be a major public health problem in Palestine. The two indigenous species *Leishmania major* and *L*. *tropica* cause most of the human CL cases. However, *L*. *infantum*, which is the main causative species of human visceral leishmaniasis (VL) in Palestine [3], was recently isolated from human CL cases as well [4]. *L*. *major* causes zoonotic CL in rodents, mainly the “fat sand rat” *Psammomys obesus*, serving as the animal reservoir. The vector is the female sandfly of the species *Phlebotomus papatasi*. However, *L*. *tropica* is considered to be anthroponotic, being transmitted from person to person by female sandfly of the species *Phlebotomus sergenti* [5, 6]. Nevertheless, recent studies from Israel have suggested that some of CL cases caused by *L*. *tropica* are zoonotic with rock hyraxes (*Procavia capensis*) serving as the animal reservoir [7, 8]. Palestinian CL cases are reported exclusively from certain foci in the Wes Bank without cases being reported in the Gaza Strip [9].

Clinical presentation of CL appears as one or multiple lesions commonly on the face, neck, arms, and legs. A papule appears at the inoculation site which may enlarge to become an indolent ulcerated nodule or plaque. Lesion presentation varies according to the causative species of the parasite. *L*. *major* frequently appears as severely ulcerated and inflamed one or multiple lesions on the skin, which usually heal spontaneously within 2–8 months. However, *L*. *tropica* appears as dry ulcers which are more difficult to treat and may persist more than a year and leaving disfiguring scars. Moreover, *L*. *infantum* typically causes a single nodular lesion of the face without appearance of crust or ulcer and without any visceral involvement. The occurrence of the three *Leishmania* species was described in many countries in the Middle East [4, 10, 11]. This occurrence is climate-sensitive. Changes in average temperature, humidity and rainfall have strong influence on vectors and reservoir hosts of *Leishmania* by influencing their survival and altering their distribution and population sizes. Fluctuations in temperature affect the developmental cycle of *Leishmania* promastigotes in the salivary glands of sand flies allowing parasites transmission and emergence of new endemic foci. Moreover, climatic hazards and disasters can lead to massive displacement and migration of population to Leishmania endemic areas thus contributing to increase incidence of the disease [12, 13].

Recent WHO reports on cutaneous leishmaniasis in the Palestinian territory highlight the need for more comprehensive studies on CL incidence, prevalence and distribution especially from Gaza Strip where no cases of human CL were reported, though CL is abundant in the nearby Sinai Peninsula [14, 15].

Population density of phlebotomine sand fly in Palestine has not been thoroughly studied. Sawalha *et al*. [16] described five potential *Phlebotomus* vectors of leishmaniasis in the West Bank of which *P*. *papatasi* constituted approximately 90%, *P*. *syriacus* 8%, *P*. *mascitti* 2%, followed by *P*. *sergenti* and *P*. *tobbi* which were less abundant. However, they concluded that *P*. *papatasi*, *P*. *sergenti*, and *P*. *syriacus* are the most probable regional vectors for *L*. *major*, *L*. *tropica*, and *L*. *infantum*, respectively. In Hebron Governate *P*. *syriacus* and *P*. *tobbi* were abundant with 45% and 10%, respectively, compared to 15% *P*. *tobbi* and 13% *P*. *syriacus* in Jenin and 45% *P*. *perfiliewi* in the north of Palestine. These species are considered as *L*. *infantum* vectors in the Mediterranean basin [3, 13].

This polymorphic and complex occurrence of CL in Palestine, which includes different modes of transmission, reservoir hosts, and *Phlebotomus* sandfly vectors is influenced by several factors. These factors include seasonal occurrence of the affecting sandfly vectors [17], which increases from mid-spring through to mid-autumn [16, 18], temperature, rainfall, humidity, and sunshine [14, 19, 20]. Moreover, CL dynamics have changed in response to environmental, agriculture, demographic and human behavioral factors. This has led to a change in the geographic distribution of the transmitting vectors and animal reservoirs.[4, 9, 19, 21].

Many infectious diseases, including leishmaniasis exhibit heterogeneity in spatial and temporal distribution [22–24]. Spatial statistical methods, based on the geographical information system (GIS), have been widely applied in the surveillance and the evaluation of the effectiveness of preventive interventions. Spatiotemporal analysis provides an efficient tool to visualize the epidemiological data and help observe the geographical distribution of disease from a more intuitive point of view. Moreover, it can identify spatial and temporal clusters from a deeper level and determine the high- and low-risk areas.

The aim of this study was to investigate eco-epidemiological parameters and spatiotemporal projections and clustering of CL in Palestine over a 30-years period from 1990 through 2020. Our study investigated demographic characteristics of reported patients by the Palestinian Ministry of Health (PMoH), CL prevalence in the Palestinian population, geographical and seasonal distribution, distribution of cases by age and gender. Furthermore, possible factors associated with CL transmission and distribution are discussed. Moreover, we investigated the spatiotemporal distribution of CL in a future projection to shed more light on the expansion potential of the disease in Palestine until 2060 based on scenarios of climate change that influence the occurrence and density of the sand fly.

## Materials and methods

### Study design

A long-term descriptive epidemiological study was conducted to investigate human CL covering the 30-year period from 1990 to 2020 in Palestine. We collected CL patient’s profiles from the primary health care clinics and annual reports as recorded by the Palestinian Ministry of Health (PMoH). The data includes age, gender, geographical origin, date of infection, diagnosis methods, and treatment. Since CL is a reportable disease in Palestine, diagnosis and treatment are provided exclusively and free of charge by the PMoH and its primary health care clinics. There was no change in the reporting system over the time period. Inclusion criteria included all patient’s profiles available in the PMoH reports and archives until 2020.

Diagnosis was based on clinical picture, microscopic examination of giemsa-stained smears of skin tissue for amastigotes, and patient’s place of residence or documented traveling to an endemic area. Serological and molecular approaches for diagnosis and *Leishmania* species identification have been introduced to the PMoH after 2010 and have led to increased specificity and sensitivity of the diagnosis.

### Study area and population

We investigated human CL cases reported by the PMoH in all eleven governorates of the West Bank: Jenin, Tulkarem, Nablus, Tubas, Jericho, Salfit, Qalqilya, Ramallah, Jerusalem, Beithlahem and Hebron; with no reported cases in the Gaza Strip.The west bank is bordered by the Kingdom of Jordon to the west. It is occupying 5640 km^2^ (including East Jerusalem) with a total population of 2.85 Mio inhabitants as reported by the Palestinian Central Bureau of Statistics (PCBS) in mid-2017 [25]. The inhabitants are distributed across 11 governorates and ranged from 70,5053 in Hebron to 49,568 in Jericho.

These areas are characterized by great variations in landform and altitude (ranges from -400m below the sea level in the Dead Sea beach to 1020m in the highest point in Hebron vicinity). The climate is mostly Mediterranean. The average temperature in the mountains over the year is 7–10 degrees lower than at the dry, hot Dead Sea shoreline. The precipitation and temperature are varied according to altitude (ranges from warm and to dry hot summers, and cool to mild winters). Terrains are mostly rugged, dissected upland in western areas, flat plains descending to Jordan Valley to the east.

### Data analysis and calculations

We calculated the average annual incidence rate (AAIR) per 10^5^ population for each of the 11 governorates in the West Bank between the years 1990–2020 based on the mid-year population size for each governorate estimated by the (PCBS) [25]. At first, we calculated the annual incidence for each governorate in each year from 1990 until 2020 by dividing the number of cases in the given year and given governorate / mid-year population size for this governorate in that year*105 population. Then we calculated the average annual incidence for each governate separately. The AAIR for the West Bank was calculated as an average of the AAIR for the 11 governates. Distribution maps and frequency tables were plotted for each governorate, year and month of infection, and the average number of cases per governorate. Moreover, cases were distributed by, age, gender and season of infection.

### Spatiotemporal analysis and projections mapping

We conducted a correlative distribution modelling with the database of the CL cases in Palestine for the time period 1990–2020 as occurrence data. The distribution of climatically suitable zones for transmission of CL was concluded based on these data and on the biology of the sandfly.

We reviewed the literature to identify environmental and ecological factors affecting sandfly distribution [23, 24, 26, 27], eight of the 19 bioclimatic variables of Worldclim (a free climate data for ecological modelling and GIS) (http://www.worldclim.org/bioclim) [28] were selected for the current and future projections. These variables are: BIO1—Annual Mean Temperature, BIO2—Mean Diurnal Range (mean of monthly (max temp—min temp)), BIO4—Temperature Seasonality (standard deviation *100), BIO7 –Temperature Annual Range (BIO5-BIO6), BIO10—Mean Temperature of Warmest Quarter, BIO11 –Mean Temperature of Coldest Quarter, BIO12—Annual Precipitation, and BIO15 –Precipitation Seasonality (Coefficient of Variation). All climate data have spatial resolution of 2.5 arc-minutes (approximately 5 km). The geographical location of each endemic village (longitude, latitude) was obtained using GeoLocator (http://tools.freeside.sk/geolocator/) (version 1.35).

We applied four modelling algorithms to model the distribution of CL in Palestine. These algorithms are: GLM (Generalized Linear Model) [29], GBM (Generalized Boosted Model) [30] and RF (Random Forest) [31] included in the biomod2 R-Package version 3.3–7 (https://CRAN.R-project.org/package=biomod2) [32] and MAXENT (Maximum-Entropy-Modelling) [33]. Ensemble modelling [34, 35] was applied using these four algorithms with each algorithm having the same weighting (25%). The available CL data from 1990–2020 were used to calculate the current and the future projections (2041–2060) for CL in Palestine. The calculation of CL future projections was performed based on mpi-esm-lr climate model and the RCP 4.5 emission scenario. The latter is an intermediate scenario expecting a raise in the global mean surface temperature between 1.1°C and 2.6°C until the end of this century [36]. RCP is the Representative Concentration Pathways (RCPs), which are used for making projections based on factors driving the Anthropogenic Greenhouse Gas emissions which include population size, lifestyle, economic activity, land use patterns, energy use, technology and climate policy. (RCPs) describe four different 21^st^ century pathways of Greenhouse Gas emissions. These include a stringent mitigation scenario (RCP2.6), two intermediate scenarios (RCP4.5) and (RCP6.0) and one scenario with very high Greenhouse Gas emissions (RCP8.5). We used GIS software (QGIS 2.8.4-Wien, http://www.qgis.org/de/site/) for mapping of the derived current and future projections of CL in Palestine.

### Ethics statement

The study used de–identified data and all information regarding individuals was made anonymous to investigators from the source (PMoH) prior to analysis. Informed consent was not necessary because the data were anonymized. This process was approved by the Research Ethics Committee at Al-Quds University. All data are available from PMoH archives and reports (http://site.moh.ps/index/index/Language/ar).

## Results

We collected and investigated the Palestinian ministry of health reports and the available CL patient’s profiles for the period between 1990–2020. The number of CL patients reported during this period was 5855 cases, and the average annual incidence rate was 18.5 cases/10^5^ population. All CL patients came exclusively from the West Bank with no cases reported in the Gaza Strip. The male to female ratio was 1.25:1.

Distribution of patients-age at onset of illness ranged from 2 months to 89 years (mean = 22.5, std 18.67 years, and the median was 18 years). 57% of the patients were younger than 20 years, 16% aged between 21–30 years old, 9% were 31–40, and 18% were 41 years or older.

**Fig 1:**
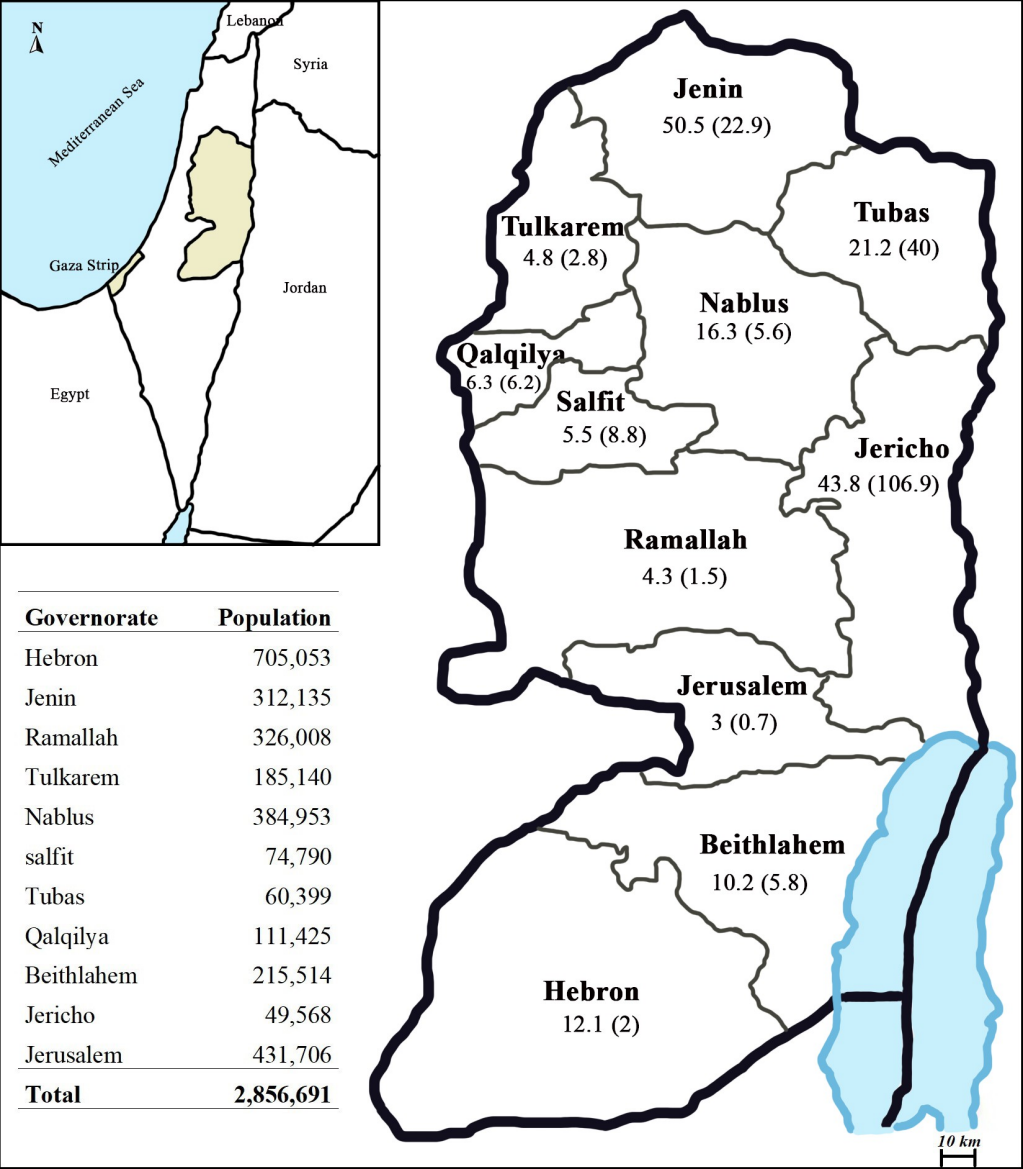
Distribution map of human cutaneous leishmaniasis in Palestine 1990–2020. The location of Gaza Strip and West Bank, Palestine (top left). Geographical distribution of human cutaneous leishmaniasis between 1990 and 2020 in each endemic governorate in the West Bank. No cases were reported from Gaza Strip. CL cases are represented by the average number of patients/year, and in parenthesis the average annual incidence rate/100.000 population calculated for each governorate from 1990 until 2020. Table 1 shows the mid-year population as estimated by the Palestinian Central Bureau of Statistics PCBS in 2017. Maps were created, adapted and edited from the freely available sources (http://eol.jsc.nasa.gov/sseop/clickmap/) by Adobe Photoshop CC 2021.

More than 65% of the cases came from three governates; Jenin 29% (1617 cases), Jericho 25% (1403), and Tubas 12% (658). The AAIR/10^5^ population and the average number of patients/year in these governates were 22.9 (50.5), 106.9 (43.8), and 40 (21.2), respectively. (Table 1) and (Fig 1) show all endemic governates in the West Bank with respective distribution of patients represented by total and average numbers per year and the AAIR/10^5^ population. No cases were reported throughout Gaza Strip during this study.

**Table 1:** Distribution of cutaneous leishmaniasis cases in the West Bank between 1990 and 2020.

Governorate	Number of patients ^a^	Percentage	AAIR/10^5^ population ^b^	Average number of patients/year
Jenin	1617	29%	22.9	50.5
Jericho	1403	25%	106.9	43.8
Tubas	658	12%	40	21.2
Nablus	521	9%	5.6	16.3
Hebron	386	7%	2.0	12.1
Beithlahem	325	6%	5.8	10.2
Qalqilya	202	4%	6.2	6.3
Salfit	176	3%	8.8	5.5
Tulkarem	148	3%	2.8	4.8
Ramallah	138	2%	1.5	4.3
Jerusalem	81	1%	0.7	3.0
**Total**	**5655**	**100%**	**18.5**	**176.7**

^a^ The total number of patients recorded by the PMoH between 1990 and 2020 in each endemic governorate

^b^ The Average Annual Incidence Rate (AAIR) per 100.000 population.

**Fig 2:**
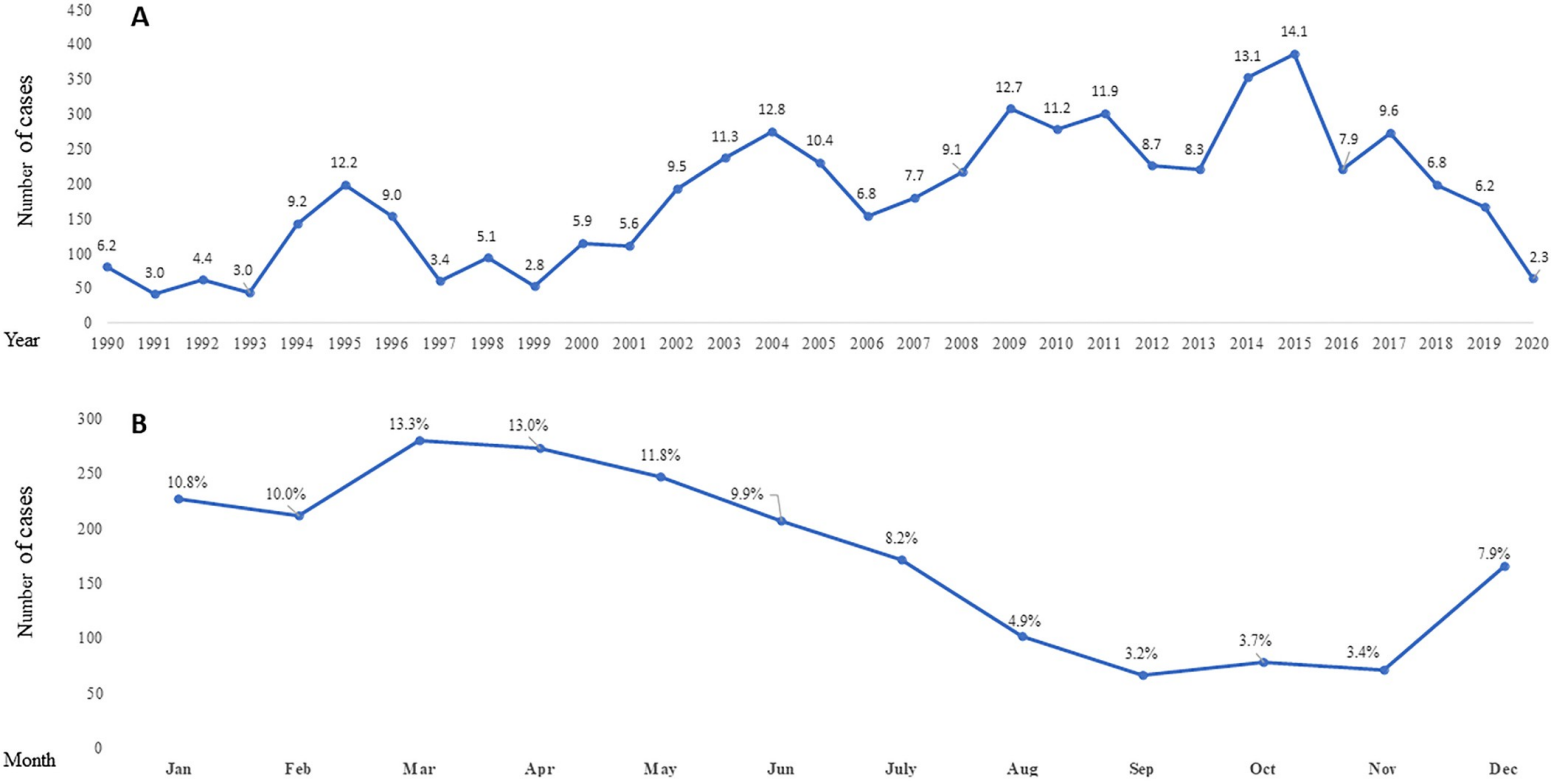
Annual and seasonal distribution of human cutaneous leishmaniasis in Palestine between 1990–2020. **(A)**: Annual distribution of cutaneous leishmaniasis patients 1990–2020. Data labels show annual incidence rate per100.000 population calculated for each year **(B):** Seasonal distribution of patients per month represented by the date of reporting as obtained from PMoH profiles. Data labels show percentage of cases per month over the studied period.

Annual and monthly distribution of CL cases were investigated over the studied period. A peak of disease incidence occurred in 1995 followed by several peaks in 2004, 2009, 2011 and the highest was in 2015. The disease peaked again in 2017 and followed by a dramatic decrease during the years after (Fig 2A). Trend of seasonality over the 30-years period was explored according to the monthly distribution of CL cases. Number of cases starts to increase on December and continues to peak during March and April of the following year. Gradual decrease of disease incidence starts on May and continues decreasing during summer season (June-August) to reach its minimum during autumn (September-November) (Fig 2B).

For 1649 patients, the expected infection date was provided together with the recording date. The average number of days between infection and recording dates for this cohort was 79 days and the median was 60 days.

Treatment of CL in Palestine is available exclusively and free of charge by the PMoH. This include intramuscular pentavalent antimonial sodium stibogluconate (Pentostam) (10–20 mg/kg body weight) daily for 10–20 days. No resistant CL cases were reported and complete healing was observed after therapy. Spatiotemporal analysis based on climatic settings in Palestine shows different climatic suitability for the sand flies transmitting CL in different parts of the West Bank and the Gaza Strip. This spatiotemporal analysis included all confirmed CL patients as reported by the Palestinian Ministry of Health over a 30-years period from 1990 through 2020.

**Fig 3:**
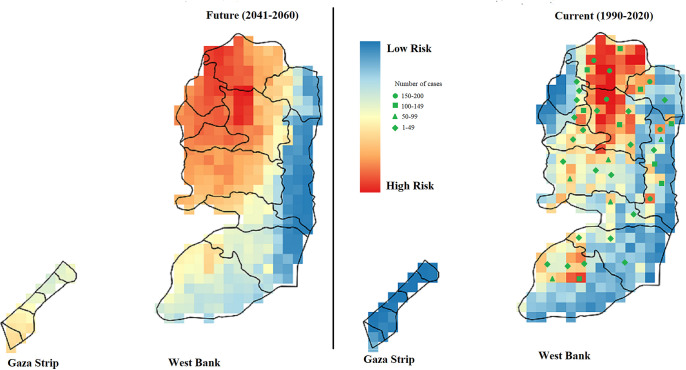
Spatiotemporal analysis of cutaneous leishmaniasis in Palestine. Spatiotemporal analysis of cutaneous leishmaniasis in Palestine for the time period 1990–2020. Designed current and future climatic suitabilities for cutaneous leishmaniasis in Palestine are shown on the maps. Current projections (right map) and the future projections (left map) are shown. Future projection of cutaneous leishmaniasis in Palestine was calculated for the coming years 2041–2060 using the mpi-esm-lr climate model and the RCP 4.5 scenario. Number of (CL) cases in each endemic area were added to the presence projections and indicated as different shapes in the map (left map), showing the number and the occurrence of CL cases in different endemic areas throughout of the country. This figure is licensed under CC BY 4.0.

The present and future climatic suitabilities for occurrence of CL for this period is shown in Fig 3. Corresponding number of CL cases in each endemic area was added as different symbols in the presence projection map (Fig 3). Moreover, we calculated the future projection for the years 2041–2060 based on expected climatic settings and their adaptability to the sandfly population. It shows higher incidences for the western West Bank. Furthermore, first cases have to be expected in the southern Gaza strip.

## Discussion

The epidemiology and spatiotemporal distribution of CL in Palestine over 30-years period were investigated. The total number of reported cases by the PMoH was 5855 and the calculated AAIR was 18.5 cases per 100.000 population. This figure did not indicate cases by causative *Leishmania* species and grouped all CL patients together. Routing diagnosis by the PMoH does not include parasite speciation and genotyping. According to previous studies [9], *L*. *tropica* and *L*. *major* are the causative *leishmania* species of CL in Palestine. However, *L*. *infantum* was isolated from few cases in the Jenin governorate [9]. CL cases was confined to the West Bank and no cases were reported from Gaza strip. Although, cutaneous leishmaniasis is abundant in North Sinai [14] and Israel [37], bordering the Gaza Strip, there is no information about *Leishmaniasis* in general from there with no autochthonous infections reported [3, 9, 23]. Like many areas in the coastal plain in Israel, the disease incidence is very low and reached to zero in many areas [37]. The most likely scenario to justify this trend of CL distribution is the ecological, climatic conditions, farming activities, cultivation, and overcrowding in Gaza Strip which are probably not preferred by the sandfly vectors nor by the reservoir host. However, misdiagnosis, self-healing, and seeking medical treatment in private clinics which do not report cases to the PMoH should not be excluded. The influence of these factors has to be thoroughly investigated and compared with the rest of the region.

Gender distribution of CL in Palestine did not show substantial differences between infected males and females. The trend of equal gender distribution was described for visceral leishmaniasis as well [3, 23]. This indicates an equal exposure of both genders to sandfly bites and equal infection with different *Leishmania* species. Age distribution of patients showed that the majority of cases ware younger than 20 years. Trends of age distribution of CL cases in Palestine remains the same over the past years as described in other studies [19].

The current geographical distribution of CL in Palestine is focused in three governorates extending from Jericho through Tubas to Jenin. However, Fig 1 shows the map of CL distribution in all governorates of the West Bank with no cases reported in Gaza Strip. Previous studies showed that *L*. *major* was significantly restricted to the Jericho district while *L*. *tropica* were from Jericho, Tubas, Nablus, Bethlehem and in all other governates [19, 37]. This unique geographical distribution of causative *Leishmania* species throughout the West Bank is determined by the sandfly populations and the parasite host distribution which are highly dependent on climatic and environmental factors. The latter showed high variability ranging from desert-like, hot, and dry environment in Jericho and Tubas in the Jordan Vally with a unique topography and elevation down to 300 m below the seas level, to hilly and mountainous districts in the middle and western parts of the West Bank.

Annual distribution of CL cases over the 30-years studied period showed multiple peaks. The first peak occurred in 1995 and most cases came from the Jericho governorate. A previous study from this area identified *L*. *major* as the causative species and attributed this peak to the socio-political changes after the Oslo agreement when thousands of Palestinian returnees from abroad and the Palestinian Police Force were resettled in Jericho governorate [19]. The second peak was in 2004 and followed by several peaks as shown in Fig 2A. Previous studies identified *L*. *major* and *L*. *tropica* as causative agents coexisting in the West Bank and contributing to theses geographical and seasonal peaks of the disease [9, 19, 37]. There are several factors contributing to the increasing CL incidence in Palestine. These include human immigration to endemic areas, urbanization and agricultural activities, land use, and climatic and environmental conditions. However, migration of CL cases in Palestine is not easy to predict and was not quantified.

Monthly distribution of the CL cases showed a seasonal pattern that trails the sandfly season and abundance, which increases from mid-spring to mid-autumn [16, 18]. The average number of days between infection and recording date was 79 days. This incubation period (~2–3 months) justifies the peak of cases from March through April following the sandfly season. However, seasonal distribution of CL cases in Jericho governate was described for *L*. *major* and *L*. *tropica* which peaks in January-February and March-April respectively [19].

Current and future projections of CL in Palestine were explored based on eight bioclimatic variables affecting climatic suitabilities of the sand fly vectors. Currant distribution based on patient’s information collected from 1990 through 2020 shows that Jericho, Tubas, Jenin and Nablus have the most suitable climatic settings for the sandfly vectors in the West Bank, while Gaza Strip was not likely the favourable. The selected bioclimatic variables including Annual Mean Temperature, Mean Diurnal Range, Temperature Seasonality, Temperature Annual Range, Mean Temperature of Warmest Quarter, Mean Temperature of Coldest Quarter, Annual Precipitation, and Precipitation Seasonality have shown high climatic suitability and favoured climatic conditions for the sand fly in these parts of the West Bank. These results are in agreement with previous study from Jericho governorate and its vicinity which found significant correlation between the monthly number of cases with mean average temperature, relative humidity, mean average evaporation, wind speed, mean average rainfall and sunshine which are favourable for the parasite vectors and hosts [19].

The future projections of CL in the West Bank until 2060 indicate a stabilization of Jenin and Nablus governorates as highly suitable regions and an increase of the suitability in the western parts of these governorates. Moreover, increasing suitability is shown in Tulkarem, Qalqilya, Salfit, and Ramallah governorates and hence a high risk of CL will expand starting from northwest of Jenin down to the southwest of Ramallah. The foci in Jericho and Tubas throughout the Jordan Vally are predicted to disappear in the future according to the projected climate changes of the bioclimatic variables included in our analysis. These changes include an increase in the annual temperature range and a decrease of the humidity to a level that cannot be tolerated by sandfly vectors. Moreover, spatiotemporal projections showed that endemic foci around Hebron city are projected to offer less favourable conditions for CL vectors and therefore are at lower level of risk. However, the western parts of Beithlahem and Hebron are projected to be at higher level of risk compared to the current situation. Interestingly, the future projection of CL until 2060 indicates an increasing suitability of climatic conditions for CL vectors in Gaza Strip, and a possible emergence of new endemics especially in the cities of Khan Yunes and Rafah to the south.

Since sandfly vector distribution depends on climatic and environmental conditions, temperature was found to be one of the most important parameters in their survival, development, behaviour, and activity [20]. Daily minimum temperature that normally recorded in the evening and at night is more vital than the daily maximum temperature since most sandfly species exhibit predominant activity periods during the night [38–41]. These activities include blood and sugar feeding, host seeking, mating, and oviposition [42, 43], However, species-specific differences in the peak activity, which can influence the vectorial capacity of different species were observed [44–46]. This can justify the differences in current occurrence and future projections between CL and VL in Palestine. Previous studies on VL indicated that the endemic foci in most governorates of the West Bank are projected to disappear in the future [23] compared to CL which is projected to continue in the West Bank and possibly emerge in Gaza Strip.

Another factor for the sandfly egg and larvae growth is humidity which originates as rainfalls and condensation. It has less important role in adulthood but is very significant in the pre-adult stages to reach the stage of a maturation of the sandfly. However, humidity in general is less important than temperature parameters [41]. Climate change associated with temperature and humidity has the potential to expand the geographic range of leishmaniasis in many countries [24, 47]. This expansion is related to a likely increase in annual maximum and minimum temperature, the latter is assumed to experience a greater increase. Moreover, precipitation in Palestine has experienced a significant decrease especially in winter and early spring. Future projections of climate changes in Palestine expect a reduction in rainfall and precipitation levels with seasonal warming and drying. On the other hand, human activities including urbanisation, agriculture and land use can contribute to changes in vegetation with subsequent changes in vector and host density and composition. Further investigations of these factors and their potentially contrasting effects are still needed.

The results of this study may have the following limitations; some cases of leishmaniasis may have been underreported because of their sub-clinical symptoms. The spatial distribution pattern and epidemiological characteristics of the disease were carried out between 1995 and 2020, data before 1995 was not available, and the environmental factors, meteorological data, urbanization, and sociological factors that may drive the leishmaniasis epidemic have yet to be examined.

In conclusion, the epidemiology and spatiotemporal distribution of CL in Palestine over 30-years period showed a climatic suitability of CL in the West Bank but not the Gaza Strip. Future projection of CL until 2060 indicates increasing incidence of CL starting from northwest of Jenin down to the southwest of Ramallah including Nablus, Tulkarem, Qalqilya, and Salfit. CL in Jericho and Tubas throughout the Jordan Vally is projected to disappear in the future and the modelling for Hebron city suggests less favourable conditions for CL vectors. In Gaza Strip, there is an increasing climatic suitability for CL vectors and a possible emergence of new endemics especially in Khan Yunes and Rafah governates. These results should be considered to implement effective control and surveillance systems in specific regions at an early stage of risk exposure. This includes periodic check of the sandfly occurrence in the areas under risk and spray living and sleeping areas of the sandfly with insecticides. Hence, the threat of the climatic-driven spatial expansion of CL vectors can be counteracted. Moreover, control of animal reservoir hosts of CL should be implemented as part of the surveillance system in the PMoH. The results of this study could improve the understanding of the epidemic characteristics and spatiotemporal patterns of leishmaniasis disease in the country. It can also aid in shaping approaches for intervention and provide a basis for policy decisions in case of health resource management and allocations in the targeted areas. On the other hand, spatiotemporal clustering analysis is a prediction strategy for a sustainable approach to monitor the spread of CL in Palestine.

## Supporting information

S1 Checklist STROBE checklist. (DOC)
