# Supplementary material for: Spatiotemporal analysis of cutaneous leishmaniasis in Palestine and foresight study by projections modelling until 2060 based on climate change prediction
Source: PLoS One. 2022 Jun 9;17(6):e0268264. doi: 10.1371/journal.pone.0268264 (PMC9182690; doi:10.1371/journal.pone.0268264)
Supplement: S1 Checklist — (DOC) [file pone.0268264.s001.doc]

STROBE Statement—Checklist of items that should be included in reports of ***cross-sectional studies***

|  | Item No | Recommendation |
| --- | --- | --- |
| **Title and abstract** | 1 | ‎The title is in the first page |
|  |  | ‎the abstract is in the second page |
| Introduction | | |
| Background/rationale | 2 | Introduction, paragraph 1, 2, 3 and 4 |
| Objectives | 3 | ‎Iintroduction, last paragraph |
| Methods | | |
| Study design | 4 | ‎Methods, paragraph 1 |
| Setting | 5 | ‎Methods, paragraph 2 and 3 |
| Participants | 6 | ‎‎Methods, paragraph 1 |
| Variables | 7 | Methods, paragraph 1 |
| Data sources/ measurement | 8* | Methods, paragraph 1 |
| Bias | 9 | (N/A) |
| Study size | 10 | Methods, paragraph 1 |
| Quantitative variables | 11 | Methods, paragraph 1  If applicable, describe which groupings were chosen and why (N/A) |
| Statistical methods | 12 | (*a*) Methods, paragraph 3 |
|  |  | (*b*) (N/A) |
|  |  | (*c*) Methods, paragraph 1 and continued in results, paragraph 1 |
|  |  | (*d*) (N/A) |
|  |  | (*e*) (N/A) |
| Results | | |
| Participants | 13* | (a) Results, paragraph 1 |
|  |  | (b) Results, paragraph 1 |
|  |  | (c) (N/A) |
| Descriptive data | 14* | (a) Results, paragraph 1 and 2 |
|  |  | (b) Results, paragraph 1 |
| Outcome data | 15* | Results, paragraph 1 and 2 |
| Main results | 16 | (*a*) Results, paragraph 2 |
|  |  | (*b*) (N/A) |
|  |  | (*c*) (N/A) |
| Other analyses | 17 | Results, paragraph 2 and 3 |
| Discussion | | |
| Key results | 18 | Discussion, paragraph 1 and 2 |
| Limitations | 19 | Discussion, paragraph 8 |
| Interpretation | 20 | Discussion, paragraph 1, 2, 3, 4, 5 and 6 |
| Generalisability | 21 | Discussion, paragraph 4,5 ,6 and 7 |
| Other information | | |
| Funding | 22 | (N/A) |

*Give information separately for exposed and unexposed groups.

**Note:** An Explanation and Elaboration article discusses each checklist item and gives methodological background and published examples of transparent reporting. The STROBE checklist is best used in conjunction with this article (freely available on the Web sites of PLoS Medicine at http://www.plosmedicine.org/, Annals of Internal Medicine at http://www.annals.org/, and Epidemiology at http://www.epidem.com/). Information on the STROBE Initiative is available at www.strobe-statement.org.
